# Unsupervised Performance Evaluation Strategy for Bridge Superstructure Based on Fuzzy Clustering and Field Data

**DOI:** 10.1155/2013/427072

**Published:** 2013-10-29

**Authors:** Yubo Jiao, Hanbing Liu, Peng Zhang, Xianqiang Wang, Haibin Wei

**Affiliations:** College of Transportation, Jilin University, Changchun 130025, China

## Abstract

Performance evaluation of a bridge is critical for determining the optimal maintenance strategy. An unsupervised bridge superstructure state assessment method is proposed in this paper based on fuzzy clustering and bridge field measured data. Firstly, the evaluation index system of bridge is constructed. Secondly, a certain number of bridge health monitoring data are selected as clustering samples to obtain the fuzzy similarity matrix and fuzzy equivalent matrix. Finally, different thresholds are selected to form dynamic clustering maps and determine the best classification based on statistic analysis. The clustering result is regarded as a sample base, and the bridge state can be evaluated by calculating the fuzzy nearness between the unknown bridge state data and the sample base. Nanping Bridge in Jilin Province is selected as the engineering project to verify the effectiveness of the proposed method.

## 1. Introduction 

With the sustained growth of road traffic and influence of external environment such as temperature, carbonation, and corrosion, the number of deteriorated bridges has increased dramatically. Their safe operation and service life are seriously threatened [1–3]. Therefore, the maintenance and repair management become particularly important. However, the human and financial resources are limited in many countries. How to determine the optimal maintenance program is critical in practice. With the development of systems theory and computer technology, bridge management system has been widely used. In order to achieve an appropriate management, the performance condition for deteriorated bridge must be evaluated [4–8].

The methods that are used for condition evaluation include existing national evaluation norms, analytic hierarchy process (AHP), and neural networks and fuzzy logic. The most widely used code in China for bridge condition assessment is “Code for maintenance of highway bridges and culvers” [9]. This code divides the bridge components into seventeen parts. Based on severity, influence degree, and changes of damage condition, the grade can be evaluated for each substructure using cumulative scores. The technical condition of entire bridge can be assessed by considering weights of seventeen parts. Sasmal and Ramanjaneyulu [10] developed a systematic procedure and formulations for condition evaluation of existing bridges using analytic hierarchy process in a fuzzy environment. Kawamura et al. [11] presented a novel approach for developing a performance evaluation system for concrete slabs of existing bridges based on neural networks and fuzzy inference. The numerical examples and conclusions reveal that the proposed approach demonstrates real potential for practical applications. Tarighat and Miyamoto [12] introduced a new fuzzy method to deal with uncertainties from inspection data, which was practically based on both subjective and objective results of existing inspection methods and tools. Wang and Elhag [13] proposed a fuzzy group decision making (FGDM) approach for bridge risk assessment. Case study revealed that the FGDM approach was a flexible, practical, and effective way of modeling bridge risks.

However, there are some drawbacks for these methods in practical application. Firstly, the adoption of evaluation index system and selection of indicators are not specific. Secondly, the scoring process for indicators is affected by subjective factors especially in existing norms. Thirdly, the determination of membership function for widely applied fuzzy logic system is difficult. Therefore, reduction of uncertainties from subjective factors is significant for improving the effectiveness in the process of evaluation.

Since state assessment methods of the existing bridge are easily influenced by subjective factors, this paper proposes an unsupervised bridge superstructure state assessment method based on fuzzy clustering according to bridge field measured data. Practical engineering is presented to verify its feasibility.

## 2. Theoretical Background

### 2.1. Theory of Fuzzy Clustering

Traditional sample classification method belongs to supervised learning style which realizes the classification through specific standards. However, fuzzy clustering method can conduct the process based on properties of the sample characteristics, and it is unsupervised. The criterion for classification is not consistent and possesses apparent dynamic characteristics. It can establish the uncertainty description of samples and more precisely reveals the actual situation [14–16].

*(1) Standardization for Clustering Data.* *x* = {*x*_1_, *x*_2_,…, *x*_*n*_} is the vector of data for classification, and each data possesses *m* properties, and *x*_*i*_ can be represented by

(1) $$\begin{array}{c} x_{i}=\left[x_{i1},{x_{i}}_{2},\ldots ,{x_{i}}_{m}\right]. \end{array}$$

An original data matrix can be constructed as follows:

(2) $$\begin{array}{c} X=\left[\begin{array}{cccc} x_{11} & x_{12} & \cdots & x_{1m}\\ x_{21} & x_{22} & \cdots & x_{2m}\\ \cdots & \cdots & \cdots & \cdots \\ x_{n1} & x_{n2} & \cdots & x_{nm} \end{array}\right], \end{array}$$

where *x*_*ij*_ is the *j*th property of the *i*th classification object.

The normalized matrix *X*′ can be calculated from the following equation:

(3) $$\begin{array}{c} x_{ij}^{\prime }=\frac{x_{ij}-\min_{1\leq i\leq n}\left(x_{ij}\right)}{\max_{1\leq i\leq n}\left(x_{ij}\right)-\min_{1\leq i\leq n}\left(x_{ij}\right)}\;j=1,2,\ldots ,m. \end{array}$$

*(2) Construction of Fuzzy Similarity Matrix.r*_*ij*_ is the similarity degree between *x*_*i*_ and *x*_*j*_; it can be calculated as

(4) $$\begin{array}{c} r_{ij}=\frac{\sum_{k=1}^{m}\left(x_{ik}-\bar{x_{i}}\right)\left(x_{jk}-\bar{x_{j}}\right)}{\sqrt{\sum_{k=1}^{m}{\left(x_{ik}-\bar{x_{i}}\right)}^{2}\sum_{k=1}^{m}{\left(x_{jk}-\bar{x_{j}}\right)}^{2}}}, \end{array}$$

where $\bar{x_{i}}=(1/m){\sum_{k=1}^{m}x}_{ik}$, $\bar{x_{j}}=(1/m){\sum_{k=1}^{m}x}_{jk}$.

*(3) Clustering Analysis.* The fuzzy similarity matrix calculated by (4) only satisfies the reflexivity and symmetry but not with transitivity. In order to conduct clustering analysis, the corresponding fuzzy equivalent matrix must be obtained. In this paper, successive square method is used to calculate the equivalent matrix as shown in

(5) $$\begin{array}{c} R^{*}=t\left(R\right)=R^{2k},\;\;R^{2k}=R^{2k-1}, \end{array}$$

where R* = *t*(*R*) and *t*(*R*) are the fuzzy equivalent matrices R*.

By selecting appropriate thresholds *λ* ∈ [0,1], the dynamic clustering map can be obtained through its truncated matrix *R*_λ_* = *t*_*λ*_(*R*).

*(4) Determination of Best Classification Threshold.* *X* = {*x*_1_, *x*_2_,…, *x*_*n*_} is the objects for classification, *x*_*j*_ = [*x*_*j*_1__, *x*_*j*__2_,…, *x*_*j*__*m*_], and *x*_*jk*_ is the *k*th feature of *x*_*j*_ (*k* = 1,2,…, *m*). *r* is the classification number corresponding to *λ*, and *n*_*i*_ is the number for the *i*th category. The average value for *k*th eigenvalue of *i*th category can be calculated as follows

(6) $$\begin{array}{c} \bar{x_{ik}}=\frac{1}{n_{i}}\overset{n_{i}}{\underset{j=1}{\sum }}x_{jk},\;k=1,2,\ldots ,m. \end{array}$$

The average value for *k*th eigenvalue of all data can be calculated using

(7) $$\begin{array}{c} \bar{x_{k}}=\frac{1}{n}\overset{n}{\underset{j=1}{\sum }}x_{jk},\;k=1,2,\ldots ,m. \end{array}$$

Assuming that *P* (*P* ≤ *n*) is the scheme number for classification, *F*-statistics analysis is used for determining the best classification threshold; it can be calculated by (8). The bigger *F* is, the better it is for classification. Consider the following equation:

(8) $$\begin{array}{c} F=\frac{\sum_{i=1}^{r}n_{i}\sum_{k=1}^{m}{\left(\bar{x_{ik}}-\bar{x_{k}}\right)}^{2}/\left(r-1\right)}{\sum_{i=1}^{r}\sum_{j=1}^{n_{r}}\sum_{k=1}^{m}{\left(x_{ik}-\bar{x_{jk}}\right)}^{2}/\left(n-r\right)}\sim F\left(r-1,n-r\right). \end{array}$$

### 2.2. Theory of Fuzzy Nearness and Approaching Principle

*(1) Fuzzy Nearness.* Given that *A* and *B* are fuzzy sets in domain *F*(*U*), denoted by *A*, *B* ∈ *F*(*U*), the inner and outer products between *A* and *B* are defined by [17]

(9) $$\begin{array}{c} A•B=\underline{\underline{\Delta }}\overset{n}{\underset{i=1}{\vee }}\left(A\left(i\right)\wedge B\left(i\right)\right),\\ A⊗B=\underline{\underline{\Delta }}\underset{x\in U}{\wedge }\left(A\left(i\right)\vee B\left(i\right)\right), \end{array}$$

where *A*•*B* and *A* ⊗ *B* are, respectively the inner and outer products, symbols ∨ and ∧ are, respectively, used to obtain maximum and minimum values.

The fuzzy nearness between *A* and *B* is defined by

(10) $$\begin{array}{c} \left(A,B\right)=\frac{1}{2}\left[A•B+A⊗B\right], \end{array}$$

where (*A*, *B*) is the fuzzy nearness between *A* and *B*. 0 ≤ (*A*, *B*) ≤ 1; the bigger (*A*, *B*) is, the better the nearness between *A* and *B* is.

*(2) Approaching Principle.* Given *A*_1_, *A*_2_ ⋯ *A*_*n*_ ∈ *F*(*U*) are the fuzzy sets. For fuzzy set *B*, if ∃*i* ∈ {1,2,…*n*}, (*B*, *A*_*i*_) = max⁡_1≤*j*≤*n*_(*B*, *A*_*j*_), then the nearness between *A*_*i*_ and *B* is much better, and fuzzy set *B* can be classified into fuzzy set *A*_*i*_.

## 3. Technical Route of Bridge Condition Evaluation Using Fuzzy Clustering

**Figure 1:**
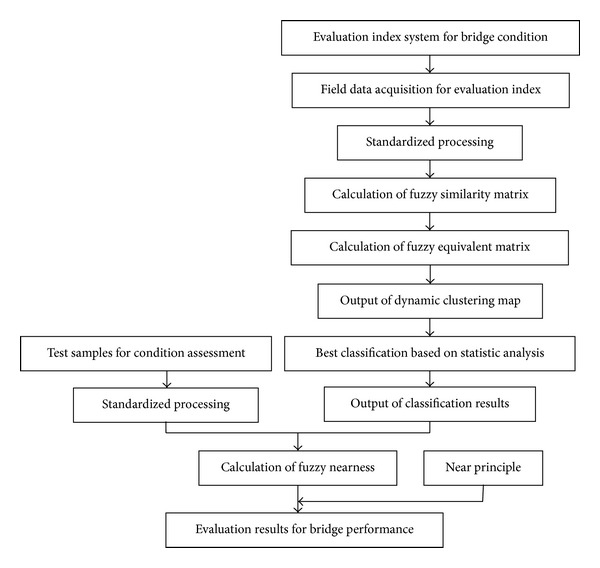
Calculation process of bridge condition evaluation based on fuzzy clustering and field data.

The technical route used in this paper for condition evaluation of bridge by fuzzy clustering is shown in Figure 1.

## 4. Fuzzy Clustering-Based Method for Bridge Condition Evaluation

### 4.1. Construction of Index System for Bridge Condition Evaluation

**Figure 2:**
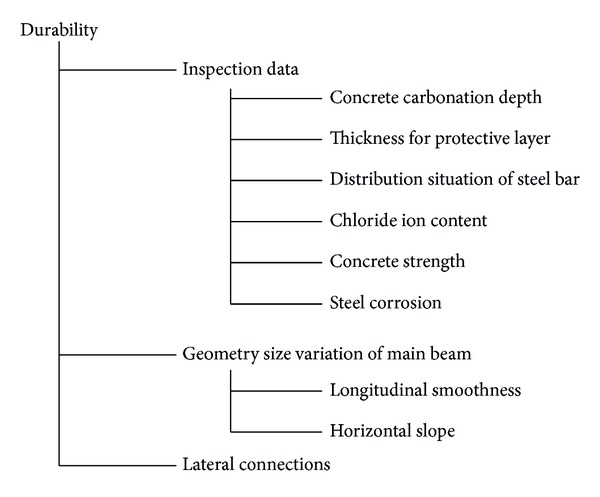
Index system for condition evaluation of bridge superstructure.

In the process of condition evaluation, the index system must scientifically, rationally, and objectively reflect the actual working status of the bridge. The index system for medium and small span bridge is determined based on the principle of integrity, simplicity, objectivity, and representativity in this paper, and it is shown in Figure 2.

### 4.2. Construction of Evaluation System Based on Fuzzy Clustering

The relative depth of carbonation calculated by (11) is used as evaluation index for degree of concrete carbonation

(11) $$\begin{array}{c} C_{d}=\frac{x_{c}}{x}, \end{array}$$

where *x*_*c*_ is the average depth of carbonation for bridge members, *x* is the average thickness of the reinforced protective layer.

Relative value of the protective layer thickness calculated by (12) is treated as evaluation index of protective layer thickness parameter

(12) $$\begin{array}{c} P_{t}=\frac{p_{m}}{p_{d}}, \end{array}$$

where *p*_*m*_ is measured value, while *p*_*d*_ is design value.

The evaluation index for distribution situation of steel bar is shown in (13). The spacing between the rebars can be measured through electromagnetic induction technology

(13) $$\begin{array}{c} S_{d}=\frac{s_{m}}{s_{d}}, \end{array}$$

where *s*_*m*_ is the measured value for steel bar spacing, while *s*_*d*_ is design value.

The percentage of chloride ion content in cement content is used as evaluation index of chlorine ion parameters; it is denoted by *C*_*l*_.

The evaluation index for concrete strength is calculated by

(14) $$\begin{array}{c} K_{bm}=\frac{R_{im}}{R}, \end{array}$$

where *R*_*im*_ is average value which can be measured by core testing, while *R* is the design strength of concrete.

Minimum value of steel corrosion potential is treated as evaluation index of steel corrosion parameters; it is denoted by *R*_*c*_.

The percentage of deviated measured points in the total points (*L*_*R*_) is used as evaluation index for longitudinal smoothness.

The percentage of offset value that exceeds ±0.3% in the total points (*T*_*R*_) is used as evaluation index for transverse slope.

Crack width of joint concrete (*C*_*w*_) is adopted as the evaluation index for lateral connections.

The inspection data for ten bridges are selected as clustering samples for condition evaluation; these samples should possibly cover every bridge condition. They are listed in Table 1.

The calculated fuzzy equivalence matrix based on fuzzy clustering theory is listed in Table 2.

Different thresholds *λ* are adopted for clustering analysis; its dynamic process is listed in Table 3.

Firstly, we determined the effective classification quantity (3, 4, 5, and 6), and *F*-statistics analysis is used to determine the best classification. The calculation results of *F*_0.05_ and *F* are listed in Table 4.

As can be seen from Table 4, when *λ* = 0.9684, the gap between *F* and *F*_0.05_ is the largest. Therefore, four categories are the best classification; the detailed results are {2, 4, 5}, {3, 6, 9}, {1, 8}, and {7, 10}.

The durability condition can be determined combined with service time of bridge; the year of opening for each bridge are listed in Table 5.

As can be seen from Table 5, the service time for {2, 4, 5} is the shortest; therefore, its condition is “very good”. Similarly, {3, 6, 9} is “good,” {1, 8} is “ordinary,” and {7, 10} is “poor.”

### 4.3. Engineering Verification

Ten samples for durability evaluation are classified into four categories, and their conditions are determined through service time. The clustering results can be treated as database for assessment; the average value corresponding to each index is regarded as the center of this category as shown in Table 6, and the other bridges can be evaluated based on approaching principle through calculation of fuzzy nearness.

**Figure 3:**
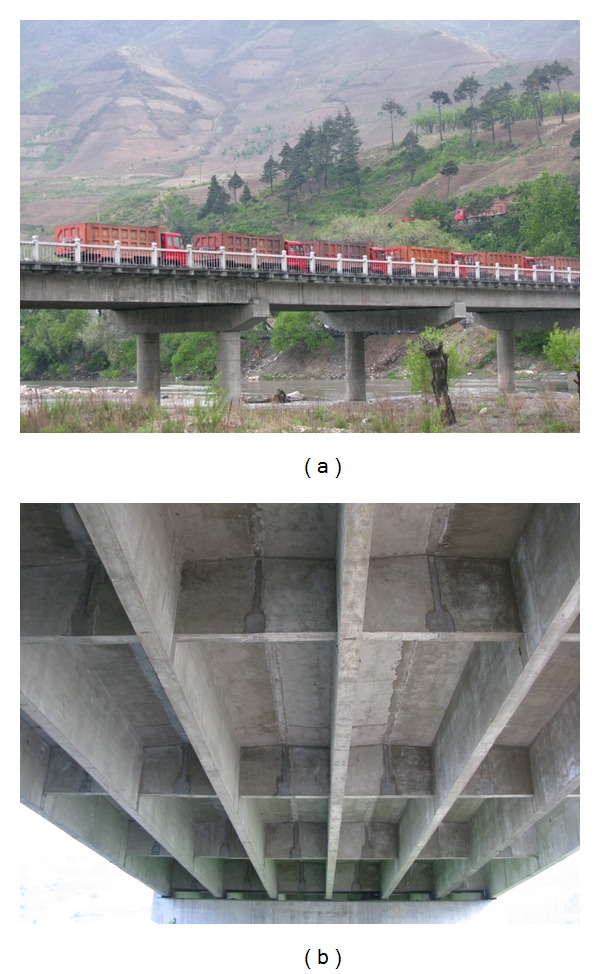
Overview of Nanping Bridge.

Nanping Bridge was built in 2005; there are totally eight spans, and they are simply supported T-beam bridges. Its overview is shown in Figure 3. The inspection data are listed in Table 7.

The calculation results of fuzzy nearness between field data of Nanping Bridge and category center for durability evaluation are listed in Table 8.

**Figure 4:**
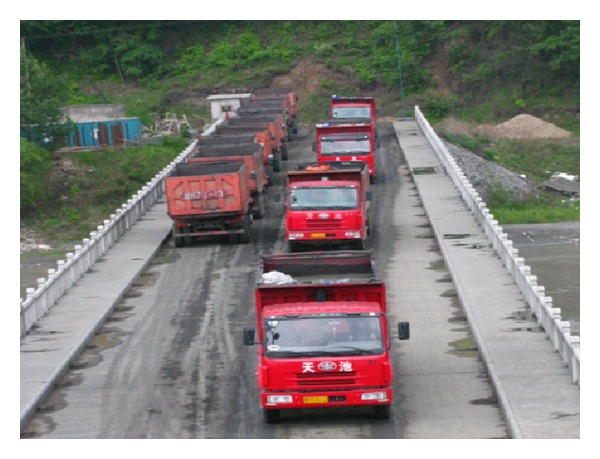
Traffic volume of Nanping Bridge.

As shown in Table 8, the fuzzy nearness between field data and “poor” is the largest. Therefore, the durability of Nanping Bridge can be evaluated as “poor”, and it needs enhancement of the conservation and maintenance. This bridge bears heavy traffic load through investigation and analysis of the traffic (Figure 4), and the evaluation result is consistent with the actual situation. It reveals that the proposed method possesses satisfactory results.

## 5. Conclusions

A fuzzy clustering-based condition assessment method is proposed in this paper. Firstly, this method builds the durability evaluation index system of bridges based on field measured parameters. And then, a certain number of bridge health monitoring data is selected as clustering samples to obtain the fuzzy similarity matrix and fuzzy equivalent matrix of samples by calculation. Finally, different thresholds are used to form dynamic clustering map and determine the best classification based on statistic analysis. The clustering result is regarded as a sample base of bridge durability state assessment. Taking the average of the corresponding indicators of the same type bridges as the approximate center of this category, this method can analyze and evaluate the bridge state for assessment on the basis of selecting the near principle by calculating the fuzzy nearness between the unknown bridge state data and the center's. Nanping Bridge is used as the physical works of the bridge durability assessment to verify the effectiveness.

The fuzzy clustering method proposed in this paper is convenient to implement. However, the disproportionality of index system is not considered. In practical engineering, each index does not have the same effect for the durability assessment of the bridge. In future work, index weight will be considered in the process of fuzzy clustering-based condition assessment of bridge.

## Figures and Tables

**Table 1:** Field data for bridge durability evaluation.

	*C*_*d*_	*P*_*t*_	*S*_*d*_	*C*_*l*_	*K*_*bm*_	*R*_*c*_	*L*_*R*_	*T*_*R*_	*C*_*w*_
1	1.2	0.79	0.97	0.52	0.93	−305	15	5	0.31
2	0.1	1.0	0.98	0.02	1.1	−12	05	0	0
3	0.7	0.87	0.96	0.31	0.97	−205	5	3	0.16
4	0.2	0.98	1.0	0.05	1.08	−35	0	0	0.02
5	0.3	0.98	0.99	0.08	1.1	−56	2	0	0.06
6	0.7	0.86	0.96	0.26	0.99	−182	6	2	0.12
7	1.8	0.62	0.95	0.88	0.90	−369	18	9	0.45
8	1.3	0.81	0.97	0.61	0.95	−256	14	7	0.36
9	0.8	0.87	0.96	0.28	0.98	−212	7	2	0.13
10	2.1	0.55	0.96	0.92	0.84	−356	20	12	0.52

**Table 2:** Fuzzy equivalence matrix for durability evaluation index system.

1	0.5732	0.5732	0.5732	0.5732	0.5732	0.9059	0.9737	0.5732	0.9059
0.5732	1	0.8836	0.9885	0.9920	0.8836	0.5732	0.5732	0.8836	0.5732
0.5732	0.8836	1	0.8836	0.8836	0.9933	0.5732	0.5732	0.9933	0.5732
0.5732	0.9885	0.8836	1	0.9885	0.8836	0.5732	0.5732	0.8836	0.5732
0.5732	0.9920	0.8836	0.9885	1	0.8836	0.5732	0.5732	0.8836	0.5732
0.5732	0.8836	0.9933	0.8836	0.8836	1	0.5732	0.5732	0.9942	0.5732
0.9059	0.5732	0.5732	0.5732	0.5732	0.5732	1	0.9059	0.5732	0.9684
0.9737	0.5732	0.5732	0.5732	0.5732	0.5732	0.9059	1	0.5732	0.9059
0.5732	0.8836	0.9933	0.8836	0.8836	0.9942	0.5732	0.5732	1	0.5732
0.9059	0.5732	0.5732	0.5732	0.5732	0.5732	0.9684	0.9059	0.5732	1

**Table 3:** Dynamic clustering results using different thresholds.

λ	Clustering results
0.5732	{1, 2, 3, 4, 5, 6, 7, 8, 9, 10}
0.8836	{2, 3, 4, 5, 6, 9}, {1, 7, 8, 10}
0.9059	{2, 4, 5}, {3, 6, 9}, {1, 7, 8, 10}
0.9684	{2, 4, 5}, {3, 6, 9}, {1, 8}, {7, 10}
0.9737	{2, 4, 5}, {3, 6, 9}, {1, 8}, {7}, {10}
0.9885	{2, 4, 5}, {3, 6, 9}, {1}, {8}, {7}, {10}
0.9920	{2, 5}, {4}, {3, 6, 9}, {1}, {8}, {7}, {10}
0.9933	{2}, {4}, {5}, {3, 6, 9}, {1}, {8}, {7}, {10}
0.9942	{2}, {3}, {4}, {5}, {6, 9}, {1}, {8}, {7}, {10}

**Table 4:** *F*-statistics calculation results for each program.

Classification quantity	3	4	5	6
λ	0.9059	0.9684	0.9737	0.9885
*F*	7.44	107.3	66.28	74.86
*F*_0.05_	4.74	4.76	5.19	6.26
*F* − *F*_0.05_	2.7	102.54	61.09	68.6

**Table 5:** Opening data for bridge.

No.	1	2	3	4	5	6	7	8	9	10

Date	1996	2011	2001	2009	2006	1999	1985	1994	1996	1980

**Table 6:** Category center for durability evaluation.

Category	*C*_*d*_	*P*_*t*_	*S*_*d*_	*C*_*l*_	*K*_*bm*_	*R*_*c*_	*L*_*R*_	*T*_*R*_	*C*_*w*_
Very good	0.2	0.99	0.99	0.05	1.09	−34	2.33	0	0.03
Good	0.73	0.87	0.96	0.28	0.98	−200	6	2.33	0.14
Ordinary	1.25	0.80	0.97	0.57	0.94	−281	14.5	6	0.34
Poor	1.95	0.59	0.96	0.9	0.87	−363	19	10.5	0.49

**Table 7:** Field data for durability evaluation of Nanping Bridge.

Index	*C*_*d*_	*P*_*t*_	*S*_*d*_	*C*_*l*_	*K*_*bm*_	*R*_*c*_	*L*_*R*_	*T*_*R*_	*C*_*w*_
Field data	1.2	0.92	0.90	0.8	0.92	−286	10	10	0.96

**Table 8:** Fuzzy nearness between field data and category center of Nanping Bridge.

Category	Very good	Good	Ordinary	Poor
Fuzzy nearness	0.6611	0.6184	0.6816	0.8171
